# A Danish study on emergency or urgent surgery for small bowel obstruction in adults: Incidence, causes, administration of antibiotics, and infectious complications

**DOI:** 10.1016/j.sopen.2021.11.004

**Published:** 2021-12-01

**Authors:** Anders Watt Boolsen, Birgitte Brandstrup

**Affiliations:** a Department of Surgery, Holbæk Hospital, Part of Copenhagen University Hospitals, Smedelundsgade 60, DK-4300 Holbæk, Denmark; b Institute for Clinical Medicine, University of Copenhagen, Blegdamsvej 3B, DK-2200 København N, Denmark

## Abstract

**Background:**

Small bowel obstruction is potentially life-threatening; however, the incidence of surgery for small bowel obstruction is unknown, the patient characteristics are poorly described, and the triggers for giving antibiotics with possible influence on complications are unclear. The aims of this study were to fill these gaps to describe the incidence and the characteristics of patients undergoing surgery for small bowel obstruction and to identify triggers for giving antibiotics and the association with postoperative infections.

**Methods:**

From July 1, 2014, to July 31, 2015, we included adult patients undergoing surgery for small bowel obstruction at 3 hospitals representing one Danish region. We collected information on patient characteristics, diagnosis, antibiotics, and infectious complications until postoperative day 90 and survival until 1 year.

**Results:**

The 3 hospitals serve a population of 656,353 adults, and treatment is free of charge. A total of 192 patients underwent emergency surgery for small bowel obstruction in the period (incidence: 27/100,000 citizens or 1,200 operations in Denmark annually). The patients with small bowel obstruction had adhesive obstruction (62%), neoplasms (11%), or hernias (7%). A total of 83% received antibiotic prophylaxis, and triggers were preoperative elevated C-reactive protein [odds ratio (95% confidence intervals): 2.49 (1.04–5.98), *P* = .041] or resection of the bowel [3.10 (1.22–7.89), *P* = .017]. The incidence of postoperative infections was not reduced among patients receiving antibiotics.

**Conclusion:**

We found that 27/100,000 patients undergo surgery for small bowel obstruction in Denmark each year. Adhesive obstruction was the primary reason (62%). A total of 83% received prophylactic antibiotics triggered by elevated C-reactive protein or bowel resection. We found no association between antibiotic use and infectious complications.

## INTRODUCTION

Small bowel obstruction (SBO) is a common condition on the surgical wards. However, the number of patients undergoing surgery for SBO and the pathology causing SBO are sparsely described, probably because no specific surgical procedure or diagnosis code exists for SBO. Moreover, the use of antibiotics (AB) and its association with postoperative infectious complications are unknown.

SBO is a life-threatening condition caused by many different diseases. Emergency surgery is often but not always necessary and can be lifesaving, but despite surgical intervention, the morbidity and mortality following SBO remain high. A study from the UK including 1,853 patients undergoing emergency laparotomy at 35 National Health Service hospitals found an all-cause 30-day mortality risk of 14.9% (median) ranging from 3.6% to 41.7% [1]. Advanced age and American Society of Anesthesiologists physical classification (ASA) class III or higher [2] greatly increased the mortality risk. In a systematic review on the management and treatment of SBO, the causes of SBO were adhesive obstruction (60%–70%), neoplasms (5%–10%), hernias (10%–15%), Crohn disease (5%–7%), and other reasons (15%) [3].

The management of SBO is nasogastric suction to minimize the distention of the bowel, intravenous fluids to treat both hypovolemia and dehydration, and sometimes surgery to eliminate the cause. For patients with adhesive SBO, nonoperative management is reported to be successful in 65%–80% of the cases [[4], [5], [6], [7]].

The Danish Ministry of Health recommends giving antibiotics to patients with SBO in general but especially if the patient has a fever and leukocytosis [8]. This recommendation is based on a case–control study of 254 patients [9]. A guideline from Uptodate.com confirms that high-quality data to guide the management of SBO are absent and that clinical practices vary greatly [10]. However, in contrast to the Danish recommendation, the Uptodate guideline, with reference to the same study [9] as above, recommends not giving antibiotics to patients with uncomplicated SBO. No other studies were found to either support or refute the administration of antibiotics [9,10].

It seems rational that patients might benefit from antibiotic treatment if contamination of the abdominal cavity is evident, ie, patients with intestinal perforation, or if bowel resection is performed [10]. However, bacterial translocation through the mucosal barrier of the gut has been shown [11,12]. In a study from the UK, sepsis developed in 41% of the patients with confirmed bacterial translocation compared with 14% in patients without bacterial translocation. However, another study including 75 patients undergoing elective colorectal surgery found no influence on outcome despite bacterial translocation in 39% of the patients [14].

A study of 251 elderly patients undergoing urgent or emergency abdominal surgery found that only 49.5% of the patients received antibiotic prophylaxis in accordance with the hospital guideline [15].

Based on the above, we can tell that the incidence of surgery for SBO and its causes are poorly described, that the recommendations for giving AB are based on a single low-grade evidence study, and that the influence of AB for the clinical outcome is unknown. Therefore, the aims of this study were (1) to describe the incidence and causes for surgery for SBO in a Danish population, (2) to identify indicators for giving antibiotics, and (3) to analyze any association between prophylactic AB and postoperative infectious complications or death.

## METHODS

The study was approved by the Danish Data Protection Agency (REG-149-2016) and the Danish Patient Safety Authority (3-3013-1999/1).

In this retrospective, observational, multicenter study, the patients were identified through a list of surgical procedures performed at each of the 3 hospitals.

Eligible patients had radiologically verified SBO and underwent emergency or urgent surgery between July 1, 2014, and July 31, 2015, at the 3 hospitals in Region Zealand receiving major emergency surgical patients. To secure 90-day follow-up, only Danish citizens were included. *Emergency surgery* was defined as surgery without planned delay.

Excluded were patients having intra-abdominal surgery up to 30 days previously, patients receiving dialysis on a regular basis, children (< 18 years), and pregnant women.

If a patient was found eligible for inclusion more than once, only the first procedure was considered.

### Data Collection and Validation

Data were collected using the hospitals' electronic patient files and the Danish Civil Registration System in which every Danish citizen is registered with a unique number.

The data were collected by the research team consisting of physicians and medical students trained in the study protocol. Two researchers examined each patient file and registered the data in a case report form. The data were typed into 2 corresponding databases and cross-checked for disagreements. If differences were found, the supervising investigator settled the case.

The following variables were extracted: sex; age; smoking habits; alcohol habits; comorbidities; preoperative American Society of Anesthesiologists (ASA) score [2]; Sepsis-2 score [16] pre- and postoperatively; preoperative CRP and leukocyte count; time in surgery; time of day of surgery; date of surgery; the diagnosis; performance of bowel resection; antibiotics administered before, during, or after surgery; infectious complications; and mortality.

Preoperative CRP > 10, leukocyte count > 12, sepsis score ≥ 2, and bowel resection were registered as possible indicators for the administration of AB before, during, or after surgery. These parameters were chosen because the guidelines recommend the surgeon to consider giving AB in the presence of 1 or more of these indicators.

Antibiotics given were piperacillin 4 g combined with tazobactam 0.5 g and metronidazole 1.5 g. In case of allergy toward penicillin, we gave cefuroxime 1.5 g.

For the possible association between antibiotics and infectious complications, the exposure variables were defined by protocol as *preoperative AB*: administration of AB within 8 hours before surgery, *intraoperative AB*: antibiotics given in the operation room, and *postoperative AB*: antibiotics initiated any time from the end of surgery to 90 days postoperatively.

The outcome was infectious complications grade ≥ 2 on the Clavien–Dindo Classification [17] 90 days postoperatively including septicemia, pneumonia, cystitis, peritonitis, intra-abdominal abscess, and wound infection; see Table 1. Septicemia was registered as a complication only if the sepsis score increased postoperatively.

**Table 1:** Definition of registered infectious complications

*Infectious complication*^a^	*Definition*
Septicemia	Scoring > 2 on the Sepsis-2-Scale, or increasing in Sepsis-2 score pre- to postoperatively
Pneumonia	Symptoms (cough, shortness of breath, fever), positive clinical findings, and medically treated
Cystitis	Urine with nitrite or positive bacterial growth, and medically treated
Peritonitis	Clinical finding, debut intra- or postoperatively
Intra-abdominal abscess	Radiologically confirmed and medically or surgically treated
Wound infection	Antibiotic treatment, presence of pus, or need for debridement

a Only complications ≥ 2 on the Clavien–Dindo Classification are registered.

All-cause mortality was registered at 30, 90, and 365 days postoperatively.

We included time of day as a possible risk factor for adverse outcomes and divided the population into day- and nightshift, the latter ranging from 4:00 pm to 7:59 am (16 hours).

### Statistical Analysis

No power calculation could be performed because the number of patients undergoing surgery for SBO and the use of antibiotics were unknown.

Descriptive statistics analyzed the study population. Continuous data were tested for normality, and the Student *t* test or the Mann–Whitney *U* test was used as appropriate. Fisher exact test analyzed frequencies.

A multivariable logistic regression model was used to identify variables significantly associated with the administration of antibiotics. In this analysis, the following variables were included: sex, age, time of day of surgery, smoking habits, alcohol habits, and ASA score.

In addition, a multivariable logistic regression model analyzed the association between antibiotics and time of day of surgery on the development of infectious complications. In the analysis, the above parameters as well as preoperative CRP, leukocytosis, sepsis score, and bowel resection were included. When analyzing for the influence of surgery during nightshift, logically, adjustment for time of day was omitted.

All analyses were 2-sided.

## RESULTS

**Fig 1:**
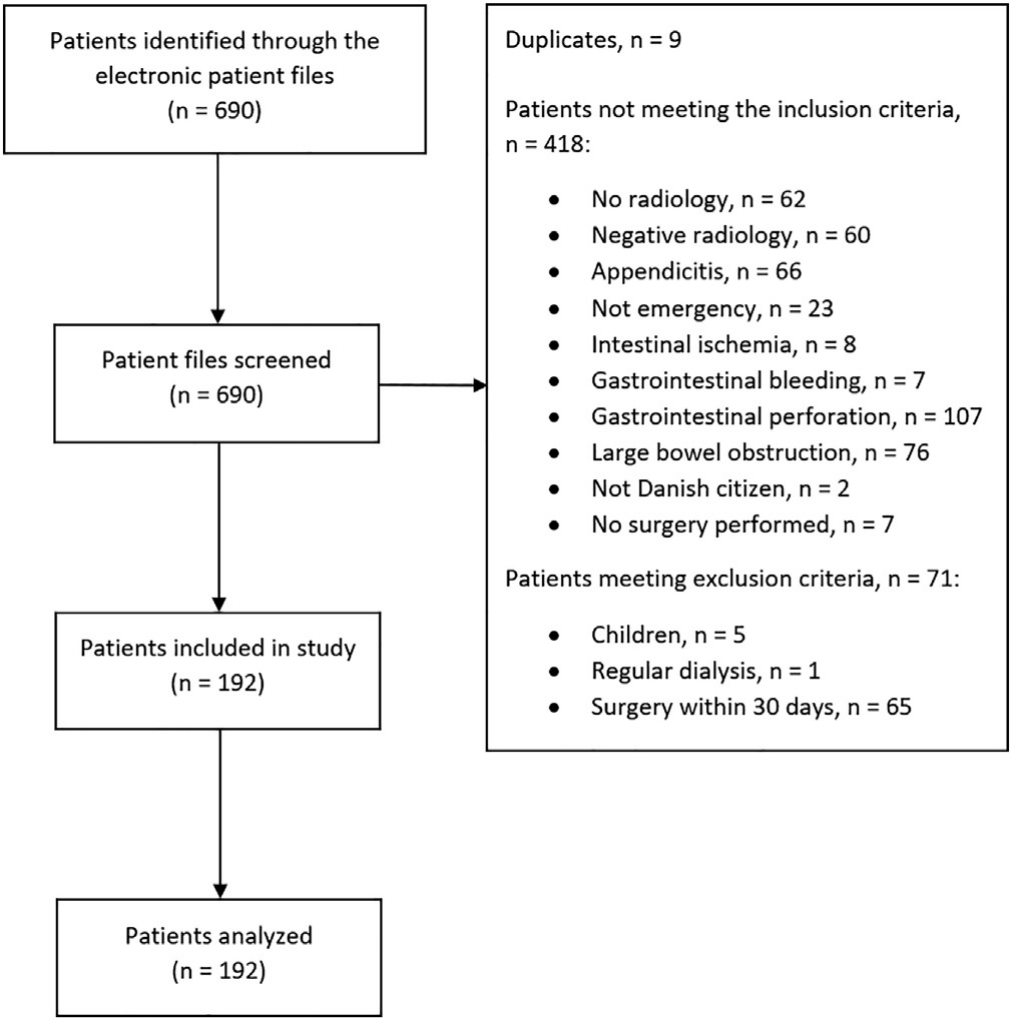
Trial profile.

A total of 192 patients were included in the study. Figure 1 shows the trial profile.

The data revealed that pre- and intraoperative AB were often distinguished by only a few minutes; therefore, the 2 groups were merged (prophylactic AB). In addition, the 14 patients that received AB after surgery were merged with the patients not given AB at all, forming 2 groups for comparison.

### Incidence of Surgery for SBO and Patient Characteristics

A total of 192 patients had surgery for SBO during the study period out of the 656,353 adult inhabitants in the Region of Zealand: incidence: 27 patients/100,000 citizens. This can be extrapolated to 1,200 operations in Denmark annually.

Table 2 shows the background and surgical data both for all the patients and for the patients receiving prophylactic AB or not. We found a tendency toward giving prophylactic AB to the patients with a higher ASA score, a diagnosis of hernia or neoplasms, or a higher alcohol intake or to current smokers.

**Table 2:** Background and surgical data

		*Received AB pre- or intraoperatively*	*Received no AB or AB after surgery only*	*Total*
Patients, *n* (%)		160 (83)	32 (17)	192 (100)
Sex, male/female, *n* (%)		69/91	14/18	83/109 (43/57)
Age, y, median (range)				70 (18–94)
Grouped by age, y, *n* (%)	18–44	16 (10)	3 (9)	19 (10)
	45–64	42 (26)	6 (19)	48 (25)
	65–84	91 (57)	21 (66)	112 (58)
	≥ 85	11 (7)	2 (6)	13 (7)
Smoking, *n* (%)	Never	66 (41)	15 (47)	81 (42)
	Current	52 (33)	8 (25)	60 (31)
	Previous	39 (24)	6 (19)	45 (23)
	NA^a^	3 (2)	3 (9)	6 (3)
Alcohol, drinks/wk, *n* (%)	0–7	132 (83)	29 (91)	161 (84)
	8–14	13 (8)	2 (6)	15 (8)
	15–21	4 (3)	0 (0)	4 (2)
	> 21	5 (3)	0 (0)	5 (3)
	NA^a^	6 (4)	7 (22)	7 (4)
ASA score,^b^*n* (%)	1	16 (10)	4 (13)	20 (10)
	2	71 (44)	21 (66)	92 (48)
	3	64 (40)	7 (22)	71 (37)
	4	8 (5)	0	8 (4)
	5	1 (1)	0	1 (1)
	6	0 (0)	0	0 (0)
Diagnosis	Adhesive obstruction	95 (59)	24 (75)	119 (62)
	Neoplasm	20 (13)	1 (3)	21 (11)
	Hernia	12 (8)	1 (3)	13 (7)
	Crohn disease	4 (3)	1 (3)	5 (3)
	Other reasons	29 (18)	5 (15)	34 (18)
Time of day for surgery	8 am–3:59 pm	58 (36)	9 (28)	67 (35)
	4 pm–7:59 am	102 (63)	23 (72)	125 (65)
Time to surgery, h, median (range)	From arrival to hospital	3.8 (1.3–30.9)	3.6 (1.3–16.6)	3.82 (0.9–30.9)
Patients operated < 6 h, *n* (%)	From arrival to hospital	129 (81)	27 (84)	156 (81)
Surgical technique, *n* (%)	Open surgery	84 (52)	24 (75)	108 (56)
	Laparoscopic surgery	12 (8)	1 (3)	13 (7)
	Converted lap. to open	63 (39)	7 (22)	70 (36)
	Missing information	1 (1)		1 (1)
Time in surgery, min, median (range)				106 (23–474)
Antibiotics given, *n* (%)	Pre-/intraoperatively			83 (43)/77 (40)
	Postop/not given			14 (7)/ 18 (9)

^*a*^Not available = not found in the medical files.

^*b*^American Society of Anesthesiologists' physical classification system.

Table 3 shows the diagnosis divided between sexes. We found no significant differences but a trend toward women having surgery for adhesive SBO more often than men.

**Table 3:** Causes of small bowel obstruction with sex information

	n *(%)*	*Male* n *(%)*	*Female* n *(%)*	*Odds for male sex* *OR (95% CI)*	*Difference between sexes* P *value*
Adhesive obstruction	119 (62)	49 (26)	70 (36)	0.41 (0.32–0.51)	.066
Neoplasms	21 (11)	8 (4)	13 (7)	0.38 (0.18–0.62)	.38
Hernias	13 (7)	8 (4)	5 (3)	0.62 (0.32–0.86)	.58
Crohn disease	5 (3)	3 (2)	2 (1)	0.60 (0.15–0.95)	1.00
Other reasons^a^	34 (18)	15 (8)	19 (10)	0.44 (0.27–0.62)	.61

a Include obstruction from biliary stones, Meckel diverticulum, volvulus, and a few cases without a specific explanation.

### The Practice for Giving Antibiotics

Part 1 of Table 4 shows the number of patients with an indicator for the administration of antibiotics divided between patients receiving prophylactic AB or not. Significantly more patients with elevated CRP or having a bowel resection received prophylactic AB compared to the patients given AB after surgery or not at all. Interestingly, a sepsis score ≥ 2 before surgery did not trigger the administration of antibiotics. One hundred sixty-five (86%) patients had at least 1 of the 4 indicators (CRP > 10, leukocyte count > 12, sepsis score ≥ 2, or bowel resection performed). Of these, 24 (14%) were not given AB, whereas 19 (10%) patients without any of the 4 indicators received AB treatment. The presence of 2 or more indicators did not change the odds of receiving AB.

**Table 4:** Indicators for antibiotic prophylaxis and postoperative infectious complications

	*AB pre- or intraoperatively* n *(%)*	*No AB or AB postoperatively* n *(%)*	*Odds ratio* *(95% CI)*^a^	P *value*^b^
All patients	160 (83)	32 (17)	0.83 (0.77–0.88)	<.001

**Part 1. Indicators for antibiotic prophylaxis**				
Preoperative CRP > 10	90 (56)	13 (41)	2.60 (1.08–6.39)	.022
Preoperative CRP missing	25 (16)	2 (6)		
Preoperative leucocyte count > 12	56 (35)	14 (44)	0.78 (0.33–1.88)	.55
Preoperative leucocyte count missing	22 (14)	2 (6)		
Preoperative sepsis score ≥ 2	52 (32)	5 (16)	2.64 (0.93–9.28)	.058
Bowel resection performed	79 (49)	8 (25)	2.9 (1.18–7.96)	.012
None of the above	19 (19)	8 (25)	0.41 (0.15–1.20)	.089
One of the above	57 (36)	13 (41)	0.81 (0.35–1.92)	.69
Two of the above	44 (28)	7 (22)	1.35 (0.52–3.97)	.66
Three of the above	28 (18)	3 (9)	2.04 (0.57–11.21)	.31
Four of the above	12 (8)	1 (3)	2.50 (0.35–110.82)	.70

**Part 2. Infectious complications**				
Superficial wound infection	20 (13)	2 (6)	2.14 (0.48–19.83)	.54
Wound infection with fascial defect	3 (2)	1 (3)	0.59 (0.046–32.09)	.52
Peritonitis	20 (13)	1 (3)	4.41 (0.65–189.25)	.21
Intra-abdominal abscess	7 (4)	0 (0)		
Septicemia	96 (60)	21 (65)	0.78 (0.32–1.85)	.69
Pneumonia	44 (28)	11 (34)	0.73 (0.30–1.81)	.52
Cystitis	23 (14)	4 (13)	1.17 (0.36–5.03)	1
Other infections	11 (7)	1 (3)	2.28 (0.31–101.60)	.69
None of the above	38 (23)	8 (25)	0.93 (0.37–2.61)	.82
One of the above	56 (35)	11 (34)	1.03 (0.43–2.54)	1
Two of the above	36 (22)	9 (28)	0.74 (0.30–1.99)	.50
Three of the above	24 (15)	4 (13)	1.23 (0.38–5.27)	1
Four of the above	6 (4)	0 (0)		
> Four of the above	0 (0)	0 (0)		

**Part 3. All-cause-mortality**				
After 30 d	17 (11)	3 (10)	1.13 (0.30–6.38)	1
After 90 d	33 (20)	4 (12)	1.65 (0.53–6.84)	.47
After 1 y	46 (28)	5 (15)	1.84 (0.66–6.38)	.28

a Odds ratio that a patient receiving AB has a specific indicator or complication calculated from frequencies.

b Calculated with Fisher exact test.

Part 1 of Table 5 shows, in a logistic regression analysis, the factors important for the prescription of AB. The analysis is corrected for age, sex, time of day, ASA score, and tobacco and alcohol habits. Because the variables CRP, leukocyte count, and sepsis scores were dependent, they were examined individually, as were bowel resections.

**Table 5:** Adjusted regression analysis of factors important for giving prophylactic antibiotics and for the development of postoperative infectious complications

	*Odds ratio (95 CI)*^a^	P *value*^a^
**Part 1. Factors important for the administration of pre- or intraoperative antibiotics**^b^		
Leukocytosis > 12	0.88 (0.37–2.08)	.78
Sepsis-2 score > 1	2.42 (0.84–6.94)	.10
Bowel resection performed	3.10 (1.22–7.89)	.017
CRP > 10	2.49 (1.04–5.98)	.041

**Part 2. Factors important for a postoperative infectious complication**^c^		
Received pre- or intraoperative antibiotics	0.76 (0.26–2.19)	.61
Surgery during nightshift	2.45 (1.04–5.81)	.041

a Calculated by logistic regression analysis.

b The analysis is adjusted for sex, 18–64 years/>64 years, dayshift/nightshift, nonsmoker/smoker, drinks 0–7/>7, and ASA 1–2/ > 3.

c The analysis is adjusted for sex, 18–64 years/>64 years, nonsmoker/smoker, drinks 0–7/>7, ASA 1–2/>3, CRP, leukocytes, and sepsis score.

Confirming the above result, preoperative elevated CRP or a bowel resection performed were the only factors significantly triggering the surgeon to prescribe AB. Neither the diagnosis (given in Table 3) nor the time of day of surgery (given in Table 2) significantly influenced the prescription practice (analysis not shown).

### Administration of Antibiotics and the Association with Postoperative Infections

Septicemia was the most predominant infectious complication. Before surgery, 57 patients (30%) had a Sepsis-2 score ≥ 2. After surgery, 117 patients (61%) had an increased Sepsis-2 score or a new infection (OR 3.6, 95% CI: 2.33–5.70, *P* < .001).

Part 2 of Table 4 shows the number of patients having a postoperative infection. A total of 146 (76%) patients had at least 1 infectious complication, and no patients had more than 4 infectious complications. We found no significant influence of prophylactic AB on the number of patients developing a postoperative infectious complication. A separate analysis of patients with adhesive SBO did not change the result (data not shown).

Prophylactic AB did not seem to influence the all-cause mortality; see part 3 of Table 4. The overall mortality was 11% after 30 days, 19% after 90 days, and 27% after a year.

Table 5 part 2 shows, in a logistic regression analysis, the factors important for the development of a postoperative infection. The analysis is adjusted for age, sexes, ASA score, smoking and alcohol habits, as well as elevated CRP and/or leukocytes and Sepsis-2 score ≥ 2. We found no significant influence of prophylactic AB on the number of patients with a postoperative infectious complication. Surgery during nightshift, however, significantly increased the odds for an infectious complication.

## DISCUSSION

We found the incidence of surgery for SBO to be 27 cases/100,000 citizens or 1,200 per year in Denmark. We were unable to find any previous studies giving an incidence of emergency surgery for SBO in Scandinavia or any other population. This is most probably because no single surgical procedure code or diagnosis code exists for SBO.

We found the practice for the administration of antibiotics to these patients among Danish surgeons to be very uneven. Only elevated CRP or resection of the bowel seemed to influence the decision of giving antibiotics or not. We could not demonstrate any influence of prophylactic AB on infectious complications following surgery.

### The Incidence, Causes, and the Differences Between Sexes in SBO

Both the incidence and the reasons for surgery for SBO may vary between countries because of differences in access to surgical care, differences in national practices (eg, incidence of women having hysterectomies), and differences in life expectancy among other reasons. In Denmark, health care is free of charge, the access to surgery is readily available, and life expectancy is relatively long, limiting the generalizability of the incidence found here.

In agreement with the finding of Rami and colleagues [3], we found adhesive obstruction to be the primary reason for SBO, followed by neoplasms, hernias, Crohn disease, and other rarer conditions. Like Miller and colleagues [18], we found women to account for 59% of the patients having surgery for adhesive SBO. In our study, the difference between sexes did not reach significance, most probably because of the small numbers. The difference might be explained by women more often having had previous abdominal surgery on the internal genitalia. In addition, infections of the internal genitalia might create adhesions and women live longer than men, thereby having a longer time to be exposed to surgery.

### The Practice for the Administration of Antibiotics in SBO

Most of the included patients received antibiotic treatment (83%), and we found that preoperative elevated CRP or the performance of a bowel resection were significant indicators. A fraction of the patients (10%) received AB without apparent indication. We found no literature addressing the surgeon's reason for giving AB. AB are recommended to patients with complicated SBO (3) or if an intra-abdominal infection or sepsis is suspected [19]; however, other factors such as ASA score, smoking and alcohol habits, or the suspicion of intestinal strangulation might influence the surgeon's decision to administer AB as well.

In our study, 86% of the patients had preoperative signs of infection or sepsis or had a bowel resection performed, thus fulfilling the criteria for giving prophylactic AB. We therefore expected the rate of AB treatment to be high. However, 14% of the study population fulfilled the criteria for receiving AB but did not. Most of the patients not receiving AB underwent surgery for adhesive SBO without bowel resection or for Crohn disease. Corrected for other important factors, the etiology of SBO did not seem to influence the decision of giving AB. The lack of existing evidence showing prophylactic antibiotic treatment to improve the outcome as presented here indeed justifies a room for variation and personal preferences.

Our results suggest that the surgical trauma itself influences the postoperative risk for SIRS and sepsis, increasing from 30% preoperatively to 61% postoperatively. We found no previous studies comparing preoperative signs of sepsis to postoperative for patients with SBO.

Somewhat unexpectedly, we found that surgery for SBO was distributed evenly throughout the day and without any difference in the use of AB. Many patients with adhesive SBO can wait for surgery until daytime (urgent surgery), and only the sickest patients are expected to have surgery during night-time and as such probably have a greater need for AB. We found that more patients operated on through the nightshift suffered a postoperative infectious complication. The reasons may be both staff and patient related: lack of sleep might increase the risk for errors in keeping the environment sterile, and sicker patients need surgery during the night. That the patients operated on in the night-time are sicker is supported by others [[20], [21], [22], [23], [24]]; however, their results contradict our findings: despite the patients being sicker, none of these studies found night-time surgery to be associated with adverse outcomes.

### Administration of Antibiotics and the Association to Postoperative Infections

We found no influence of prophylactic AB on mortality or number of patients with an infectious complication. However, in our study, not being a randomized clinical trial, a bias by indication of selecting the sickest patients for treatment with AB cannot be ruled out.

To meet this bias, we performed a separate analysis for the patients having adhesive SBO. Patients with adhesive SBO are rarely in a preoperative state of bacterial sepsis, and bowel resection for necrotic gut is not often needed. The separate analysis confirmed no difference in outcome between patients given AB or not.

Another explanation for our findings might be the timing of AB in relation to the surgical procedure. In a retrospective study of 4,453 patients undergoing general surgery, the administration of AB 4 minutes before surgery significantly reduced infectious complications compared with up to 60 minutes before surgery^25^. We analyzed AB given up to 8 hours before surgery with AB given during surgery, perhaps influencing the result.

We found an overall 30-day mortality of 11%, which is acceptable compared to the 30-day mortality of 14.9% found in the UK [1] or the 13% found in a Danish single-center study [24]. However, after a year, nearly twice as many patients who were given AB had died compared with the patients who were not given AB. This is in accordance with our finding that the patients given AB had a higher ASA score.

### Strengths and Weaknesses

The strengths of this study are the multicenter design increasing the generalizability of our results, and the careful reading of all the medical records with both double data extraction and double entry into the database, thus minimizing the risk of errors.

The retrospective design, however, can only generate hypothesis. A randomized trial is needed for giving recommendations on the continuous use of prophylactic AB to this group of patients. Moreover, the retrospective design limits the data to the ones noted in the patient files. One could want for a larger sample size, a more precise registration of the timing of giving AB, and a more even distribution of patients in the groups (given AB or not) compared. It is not possible in this study to ask the surgeon why AB treatment was commenced in one patient and not in another, and any conclusions on the effect of antibiotics on postoperative infections may be biased by indication as discussed above.

In conclusion, we estimated 1,200 patients to undergo surgery for SBO in Denmark per year (27 cases per 100,000), with adhesive obstruction as the primary reason (62%) followed by neoplasms (11%), hernias (7%), Crohn disease (3%), and other reasons (18%).

A total of 83% of the patients received prophylactic antibiotics, and administration of antibiotics was associated with preoperative elevated CRP or bowel resection, but the practice differed widely.

With reservations regarding the retrospective design, we found no association between prophylactic antibiotics and postoperative infections or all-cause mortality. The mortality was 11% after 30 days, 19% after 90 days, and 27% after 1 year.

Randomized trials to guide the use of prophylactic AB during surgery for SBO are highly needed, and this study can help generate the hypothesis and form the basis for future power calculations.

## Author Contribution

Anders Watt Boolsen: Developed the idea, searched the literature, drafted the protocol, collected the data, planned the analysis and interpretation, conducted the analysis, drafted the manuscript, and revised and approved the final manuscript.

Birgitte Brandstrup: Planned and supervised the study, refined the drafted protocol, planned the analysis, revised the analysis and interpretation, revised and approved the final manuscript, and raised the funds.

## Conflict of Interest

Both authors declare no conflict of interest.

## Funding Source

We want to thank the following funds for their unrestricted support:

1. The Department of Surgery, Holbæk Hospital, part of Copenhagen University Hospitals
2. The Region of Zealand Research fund
3. The Joint Region of Zealand and Region of Southern Denmark Research fund
4. Merchant Chr. Andersen and wife Ingeborg Andersen's fund, founded by their daughter, Miss Lilli Ellen Andersen
5. Axel Muusfeldts Fond

## Ethics Approval

The study was approved by the Danish Data Protection Agency (REG-149-2016) and the Danish Patient Safety Authority (3-3013-1999/1).
